# Transfer Time from the Intensive Care Unit and Patient Outcome: A Retrospective Analysis from a Tertiary Care Hospital in India

**DOI:** 10.5005/jp-journals-10071-23132

**Published:** 2019-03

**Authors:** Sharmila Chatterjee, Saswati Sinha, SK Todi

**Affiliations:** 1 Department of Academics and Research, AMRI Hospitals, Kolkata, West Bengal, India; 2,3 Department of Critical Care Medicine, AMRI Hospitals, Kolkata, West Bengal, India

**Keywords:** Intensive care, Length of stay, Mortality, Readmission, Transfer time

## Abstract

**Background and aims:**

Patients' outcome after ICU transfer reflect hospital's post-ICU care status. This study assessed association of after-hour ICU transfer on patient outcome.

**Subjects and methods:**

Single-centre, retrospective analysis of data between March 2016 and April 2017 was performed at a tertiary-care hospital in India. Patient data were collected on all consecutive ICU admissions during study period. Patients were categorized according to ICU transfer time into daytime (08:00-19:59 hours) and after-hour (20:00-07:59 hours). Patients transferred to other ICUs/hospitals, died in ICU, or discharged home from ICU were excluded. Only ?rst ICU admission was considered for outcome analysis. Primary outcome-hospital mortality; secondary outcomes-ICU readmission and hospital length of stay (LOS). All analysis were adjusted for illness severity.

**Results:**

Of 1857 patients admitted during study period,1356 were eligible for study; out of which 53.9% were males and 383(28%) patients transferred during after-hour. Mean age of two groups (daytime *vs.* after-hour 65.7±15.2 *vs.* 66.3±16.2 years) was similar (*p* = 0.7). Mean APACHE IV score was comparable between daytime *vs.* after-hour transfers (45.6±20.4 *vs* 46.8±22; *p* = 0.05). Unadjusted hospital mortality rate of after-hour-transfers was significantly higher compared to daytime-transfers (7.1% *vs.* 4.1%; *p* = 0.02). After adjustment with illness severity, after-hour-transfers were associated with significantly higher hospital mortality compared to daytime-transfers(aOR1.7, 95%CI 1.1,2.8; *p* = 0.04). Median duration of hospital LOS and ICU readmission though higher for after-hour-transfers, was not statistically significant in adjusted analysis (aOR^hospitalLOS^1.1, 95% CI 0.8, 1.4, *p* = 0.5; aOR^readmission^ 1.6, 95% CI 0.9,2.7; *p* = 0.06, respectively).

**Conclusion:**

After-hour-transfers from ICU is associated with significantly higher hospital mortality. Hospital LOS and readmission rates are similar for daytime and after-hour -transfers.

**How to cite this article:**

Chatterjee S, Sinha S *et al*., Transfer Time from the Intensive Care Unit and Patient Outcome: A Retrospective Analysis from a Tertiary Care Hospital in India. Indian J Crit Care Med 2019;23(3):115-121.

## INTRODUCTION

Intensive care unit (ICU) patients account for about 20 to 50% of in-hospital mortality rates^1,2^. Studies show approximately 10.8% ICU patients dying in general wards after being transferred from the ICU^3,4^. Safe and efficient transition of patients from ICU to the general ward often requires a proper transfer-out planning from the ICU^5,7^. Most deaths after ICU transfer have been attributed to higher disease severity (as evaluated with acute physiology and chronic health evaluation (APACHE) scores), older age, organ failure and do-not-resuscitate orders^3,4^. However, recent studies also demonstrate an association between ICU transfer time to wards and hospital morality rates. After-hours transfer, defined as transfer from ICU at night and out-of-office hours have been reported to be associated with adverse hospital outcomes such as higher hospital mortality^7,8^, higher ICU readmission rate^9,10^, and prolonged hospital length of stay (LOS)^10^. The excess mortality associated with after- hours transfer demonstrated in these studies also infer that the observed mortality is possibly due to premature transfer of patients or post-ICU suboptimal ward care in the after- hours^7,10^. Premature transfers are much more common at night^11^ and are generally the reflection of limited ICU bed capacity^9,11–14^, an increased demand for ICU beds or lack of nursing staff^15^.

Despite these results, some studies even failed to establish an association with after-hours transfer and hospital outcome. A Finnish multicenter study conducted in 18 ICUs did not ?nd “out-of-of?ce hour” transfer to be significantly associated with increased mortality after adjusting for illness severity, intensity of care, and presence of advanced directives (Odds ratio (OR) 1.1, 95% confidence Interval (CI) 0.93-1.3)^16^. Another study also showed night-time transfers to be associated with increased mortality in busy tertiary care hospitals^17^. Such discrepancies have been reported to be due to variations in night time definition, differences in patient population, study design and diverse local healthcare systems.

Patients' hospital outcome after ICU transfer depends upon post ICU care in the hospital and is often regarded as a quality indicator of the hospital. Furthermore, to date, there have been no studies from India that have investigated the effect of ICU transfer time on patient outcomes. We hypothesized that outcomes would be adverse for ICU patients transferred during after-hours compared to day-time transferred patients. A retrospective study was thus performed to assess the association of after-hour transfer from ICU to ward on subsequent hospital mortality, hospital LOS and ICU readmission.

## METHODS

This retrospective cohort study was conducted in a 23 bedded ICU of a tertiary care hospital in India over a 14 month period between March, 2016 and April, 2017. The study was approved by the Institutional Review Board and informed consent was waived because the study was observational in nature and involves the use of de-identified data from a pre-existing ICU database.

### Study Population

The study included all adult patients (=18 years age) consecutively admitted to the ICU with = 24 hours of ICU stay during study period, and discharged alive from ICU to wards. Patients who died in the ICU, transferred to other hospitals or nursing homes, discharged home from ICU or discharged against medical advice were excluded. Patients with missing data were also excluded. Only the first admission was considered for patients with multiple ICU admissions during a single hospitalization.

### Definition of Variables

For the purpose of this study, we defined “after-hour” transfer as transfer from ICU occurring between 08:00 pm and 07:59 am, and “daytime” transfer as transfer from ICU between 08:00 am and 07:59 pm. ICU readmission was defined as patients' getting back to ICU from wards within the current hospitalization irrespective of the time between their ICU discharge and readmission to ICU. ICU admission source was categorized into those arriving from the emergency, ward, other hospitals, operation theaters, and high dependency units (HDU). Admission diagnosis was used to classify the body system involved. Presence of any cardiovascular disease, chronic obstructive airway disease, diabetes mellitus, neurological diseases and chronic kidney diseases were the major comorbidities considered in our study. The APACHE IV score was used to determine severity of illness on admission. The APACHE IV determines the severity of illness by including a number of physiological, biochemical and demographic (e.g. age, blood pressure and chronic health status) variables to calculate a numerical score to assess the predicted risk of death^18^. Hospital LOS was defined as the total number of days that a patient stayed in hospital, including ICU stay, from the time of admission. ICU support status was determined by whether patients required invasive mechanical ventilation (IMV), non-invasive mechanical ventilation (NIMV), ionotrope/vasopressor support or blood transfusion during their ICU stay.

Other variable information collected included patient demographics and ICU discharge status (patients discharged alive or dead, whether discharged home, or to the ward, other ICUs or high dependency units (HDU), or discharged against medical advice) and hospital discharge status as discharged alive or dead.

### Data Source and Data Quality

In-patient information was extracted from the ICU database. The database regularly updated by two trained data entry operators, contain information on 220 variables of ICU patients. The data entry operators scrutinize in-patient records and investigation files daily to collect information on all relevant variables. Two full-time ICU consultants supervise the collected data daily. Any data discrepancy in the database is cross-checked with patient records for errors during entry. For this study, all patient personal identifiers were removed from data files to maintain patient confidentiality and all patients in the database were provided with a unique ID number. Quality of data was assessed by screening 50 randomly selected records every month. Any difference was resolved by consensus. All data were collected on a pre formed structured Excel data sheet.

### Statistical Analysis

Time of ICU discharge (after-hours *vs.* daytime) was the primary exposure. The primary outcomes of our study were crude and risk-adjusted in-hospital mortality and secondary outcomes were hospital LOS and ICU readmissions. Descriptive data has been expressed as mean (SD), median (interquartile range), or percentages. Student's *t* test was used to describe normally distributed data while Wilcoxon's rank sum test was used for analyzing nonparametric data. Chi-square test was used to analyze categorical data. Bivariate and multivariable logistic regression models were used to estimate crude and risk adjusted estimates for “after-hours” transfer risk, with adjustment for APACHE IV and other co-variates. Results were reported as odds ratios and 95 % con?dence interval. Two-tailed tests were used with a significance level at a =0.05. All statistical analysis was performed using the PC-SAS program (V9.2, SAS Institute, Cary, NC, USA).

## RESULTS

**Graph 1:**
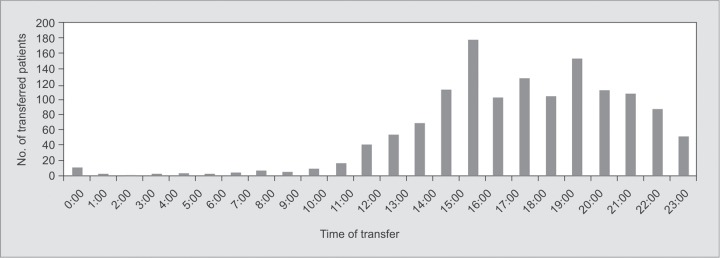
Number of patients transferred out of ICU by hour

There were a total of 1857 patients admitted during study period. After excluding patients who were discharged home from ICU directly (n = 73), or discharged against medical advice (DAMA) (n = 108) and patients who died in the ICU (n = 108), survival till ICU transfer to ward occurred in 77.5% (n = 1439) of admitted patients. Another 83 (4.4%) were excluded due to missing data and the final study population consisted of 1356 patients. Of these, 383 (28.2%) transfers occurred during after-hours, from 08:00 pm to 07:59 am, and 973 (71.8 %) were daytime transfers (from 08:00 am to 07:59 pm) (Graph 1). Sixty-nine (3.7%) patients withdrew or withheld life sustaining treatment in the ICU. While majority (92.8%) of them either expired in ICU or were discharged against medical advice, 5 patients were transferred to the ward out of which 1 patient was eventually discharged from hospital.

The mean age of patients transferred during after-hours did not significantly differ from those transferred during daytime (66.3 ± 16.2 *vs.* 65.7 ± 15.2 years, *p* = 0.7). The sex distribution of patients was also similar in the two transfer periods (male: 55.4% *vs.* 53.3%; *p* = 0.5). Significantly higher number of patients transferred during after-hours were admitted from the HDU (3.7% *vs.* 1.9%; *p*=0.05) compared to daytime transferred patients. Day- time transferred patients were more commonly admitted from the operating theaters (13.8% *vs.* 8.1%; *p* = 0.004). There was no significant difference in comorbidities between after-hour and daytime transfers (77.6% *vs.* 77%; *p* = 0.6). Mean APACHE IV scores and predicted mortality rates were significantly higher for after- hour transfer patients compared to day time transfer patients respectively (46.8 ± 22 *vs.* 45.6 ± 20.4, *p* =0.05; 9.7% *vs.*7.8%, *p* = 0.009). After- hour transfer patients were sicker patients and more likely to have received mechanical ventilation and ionotrope support (18.5% *vs.* 13.5%, *p* = 0.03; 19.3% *vs.*11.6%, *p* = 0.0003) during their ICU stay. Significantly higher number of patients with diagnosis of sepsis were transferred during after- hours (6.8% vs 2.9%; *p*=0.0009). The characteristics of all patients transferred during daytime and after-hours are described in Table 1.

**Graph 2:**
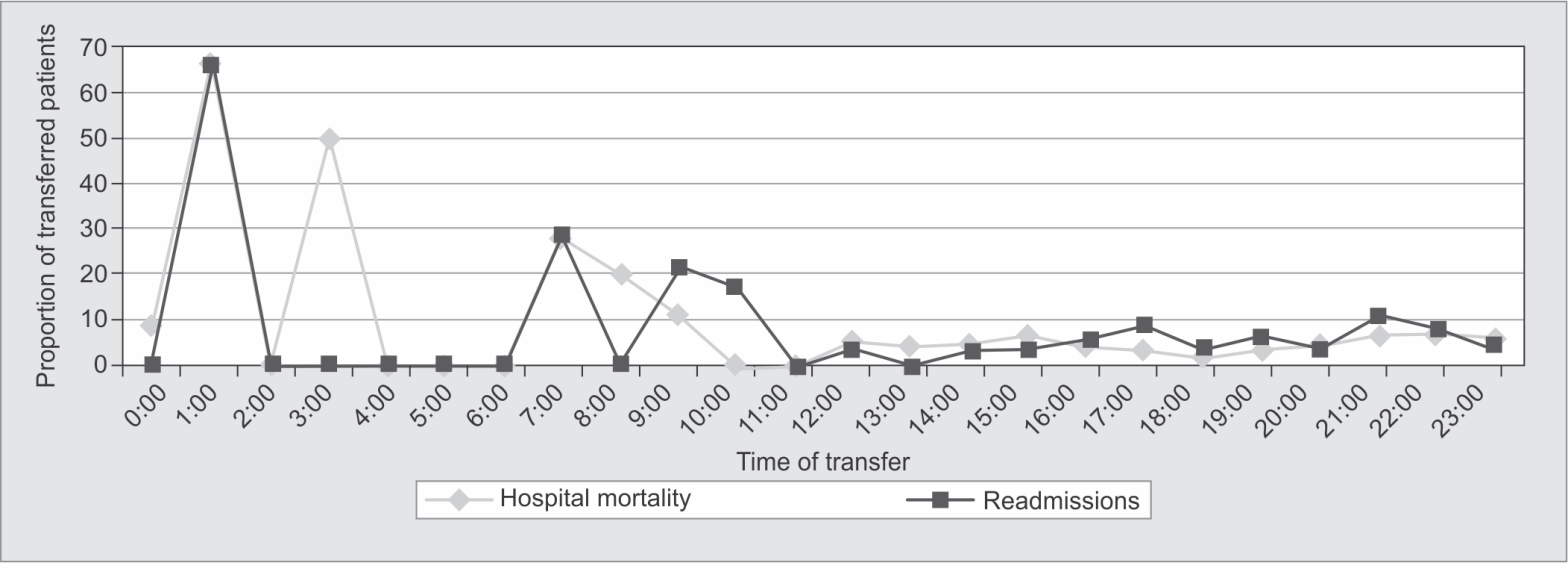
Hospital mortality and readmission rates of transferred patients by hour

Hospital mortality was significantly higher among after-hour transferred patients compared to daytime transferred patients (7.1% *vs.*4.1%, *p* = 0.02). ICU readmissions and the median LOS in hospital also differed significantly among the two transfer groups (Table 2, Graph 2).

### After hour transfer and hospital mortality, ICU readmission and hospital LOS

On bi-variate analysis, factors that were seen to be associated with increased risk of hospital mortality were higher age (OR 1.8, 95% CI 1.1-3.0, *p* = 0.02), presence of comorbidities (OR 2.5, 95% CI 1.1-5.6, *p* = 0.02), higher APACHE IV score at admission (OR 1.2, 95% CI 1.1-1.6, *p* < 0.0001), receipt of ICU support (OR 1.8, 95% CI 1.1-3.0, *p* = 0.02) and after-hour transfer (OR 1.8, 95% CI 1.1-3.0, *p*= 0.02) (Table 3). However, adjusted analysis in the multivariable logistic regression model showed that after-hour transfer remained the single most significant independent factor associated with hospital mortality (aOR 1.7, 95 % CI 1.1-2.8, *p*=0.04) (Table 4). After-hour transfer was also associated with higher odds of readmission and hospital LOS, but this was not statistically significant in the adjusted model (aOR^readmission^ 1.6, 95% CI 0.99-2.6, *p*=0.06; aOR ^hospital LOS^ 1.1 95% CI 0.8-1.4, *p*=0.5).

Of the 383 patients transferred out of the ICU at after-hours, 43 (11.2%) had an adverse outcome - death or ICU readmission. These patients significantly differed from those who were eventually discharged alive in terms of their mean age, APACHE IV scores, comorbidities, ICU support status and predicted mortality rates (PMR). Differences between the after-hour discharged patients with and without adverse outcome are listed in Table 5.

## DISCUSSION

Our study demonstrated increased mortality (crude and risk- adjusted) associated with after-hour transfer from ICU compared to daytime transferred patients. These findings are in agreement with findings from Goldfrad and Rowan in UK^11^ who first demonstrated that patients transferred during after-hours had a significantly higher crude as well as case mix adjusted mortality compared to daytime transfers (crude OR 1.46, 95% CI 1.18-1.80 and case-mix adjusted OR 1.33, 95% CI 1.06-1.65). They postulated that the increase in mortality among after-hour transfer patients was a reflection of rising demand on ICU beds leading to premature ICU discharges^11^. The adverse impact of after-hour discharges were further documented by several studies from UK^19^, USA^10^, Australia^7–9,14,19^ and Canada^15,20^. After-hour ICU discharge, not considered optimal care or standard practice, is often due to increased pressure on ICU beds to accommodate a new patient, or organizational aspects such as unavailability of a bed in the ward until late hours. Both these factors are likely to have played a role in our study as the average ICU occupancy rates are high causing premature discharges and delays in transfer occur when designated bed in the ward to which the patient is to be transferred is not vacant. Moreover less surveillance, lower nurse-to-patient ratios and decreased staff availability in the wards during after-hours could also attribute to increased mortality for patients transferred from ICU^14^. Transfers in the middle of the night may cause both physical and psychological trauma to patients^11^.

**Table 1:** Patient characteristics for daytime and after-hour transfers

*Characteristics*	*Daytime (n = 973)*	*After-hour (n = 383)*	*P*
Age, mean ± SD	65.7 ± 15.2	66.3 ± 16.2	0.7
Gender, male, n (%)	519 (53.3)	212 (55.4)	0.5
BMI^a^, mean ± SD	22.2 ± 2.7	22.2 ± 2.6	0.2
Source of admission n(%)			
Emergency	628 (64.4)	265 (69.2)	0.1
Ward	142 (14.6)	51 (13.3)	0.5
OT^b^	134 (13.8)	31 (8.1)	0.004
Other hospitals	9 (0.9)	4 (1.04)	0.8
HDU^c^	18 (1.9)	14 (3.7)	0.05
Other ICU	42 (4.3)	18 (4.7)	0.8
Admission diagnosis n(%)			
Cardiovascular	94 (9.7)	30 (7.8)	0.3
Respiratory	208 (21.4)	80 (20.9)	0.8
Gastrointestinal and hepatobiliary	194 (19.9)	66 (17.2)	0.3
Genito urinary	189 (19.4)	69 (18)	0.6
Neurological	47 (3.5)	24 (1.8)	0.3
Infectious diseases	25 (2.6)	16 (4.2)	0.2
Sepsis (all causes)	28 (2.9)	26 (6.8)	0.0009
Metabolic	64 (6.6)	28 (7.3)	0.6
Trauma	12 (1.2)	8 (2.1)	0.6
Dermatology	3 (0.3)	0	0.3
Hematology	19 (1.9)	6 (1.6)	0.2
Musculoskeletal	65 (6.7)	18 (4.7)	0.2
Operative status n(%)			
Medical	803 (82.5)	337 (88)	0.05
Comorbidities n(%)	759 (78)	297 (77.6)	0.6
Cardiovascular diseases	629 (64.6)	247 (64.5)	0.9
Diabetes mellitus	404 (41.5)	158 (41.3)	0.9
Chronic obstructive airway diseases	152 (15.6)	53 (13.8)	0.4
Chronic kidney diseases	102 (10.5)	50 (13)	0.2
Neurological diseases	89 (9.2)	45 (11.8)	0.1
APACHE IV, mean ± SD	45.6 ± 20.4	46.8 ± 22	0.05
ICU support, n(%)	344 (35.4)	169 (44.1)	0.003
IMV ^d^	134 (13.8)	71 (18.5)	0.03
NIMV^e^	102 (10.5)	51 (13.3)	0.1
Noradrenalin	113 (11.6)	74 (19.3)	0.0003
Vasopressin	3 (0.3)	4 (1.04)	0.1
Blood transfusion	171 (17.6)	66 (17.2)	0.9
Predicted mortality rate	7.8	9.7	0.009

^a.^ *BMI:* Body mass index

^b.^ *OT:* Operation theatre

^c.^ *HDU:* High dependency unit

^d.^ *IMV:* Invasive mechanical ventilation

^e^ *NIMV:* Noninvasive mechanical ventilation

**Table 2:** Outcome differences between after-hour and daytime transfers

*Outcome variables*	*Daytime (n = 973)*	*After-hour (n = 383)*	*P*
Hospital mortality, n(%)	40 (4.1)	27 (7.1)	0.02
ICU readmission, n(%)	48 (4.9)	29 (7.6)	0.05
Hospital length of stay, days median (IQR)	8 (6 - 13)	9 (6 - 14)	< 0.001

**Table 3:** Odds ratios (OR) and corresponding 95% con?dence intervals (CI) for clinical variables associated with hospital mortality after ICU transfer

*Clinical factors*	*OR*	*95% CI*	*P*
Age	1.02	1.01 - 1.04	0.01
Sex (ref = male)	0.6	0.4 - 1.01	0.06
Comorbidities	2.5	1.1 - 5.6	0.02
APACHE IV	1.2	1.1 - 1.6	< 0.0001
ICU support	1.8	1.1 - 3	0.02
Source of admission	1.02	0.9 - 1.2	0.8
Emergency	1.1	0.8 - 1.2	0.2
Ward	1.1	0.6 - 2.2	0.7
HDU	8.7	3.8 - 19.6	< 0.0001
OT	0.8	0.4 - 1.7	0.5
Other hospitals	4.1	0.9 - 10.1	0.7
Admission diagnosis			
Cardiovascular	1.2	0.5 - 2.6	0.7
Respiratory	1.1	0.6 - 2	0.7
Gastrointestinal and hepatobiliary	1.3	0.7 - 2.3	0.4
Genitourinary	1.4	0.8 - 2.5	0.2
Neurological	0.3	0.04 - 2.0	0.2
Infectious diseases	1.01	0.3 - 2	0.9
Sepsis (all causes)	1.6	0.6 - 4.6	0.4
Metabolic	1.1	0.4 - 2.8	0.8
Trauma	0.6	0.08 - 2.3	0.9
Transfer time (ref = daytime)	1.8	1.1 - 3.0	0.02

**Table 4:** (Adjusted) Multivariable logistic regression analysis showing the independent association of transfer time with hospital outcome

*Predictor variable - Time of transfer*	*Outcome variable*	*OR*	*95% CI*	*P*
Daytime	Hospital mortality	Reference		
After-hour		1.7	1.1 - 2.8	0.04
Daytime	ICU readmission	Reference		
After-hour		1.6	0.99 - 2.7	0.06
Daytime	Hospital LOS	Reference		
After-hour		1.1	0.8 - 1.4	0.5

**Table 5:** Differences between after-hour transfers with and without adverse outcome

*Age, comorbidities and severity of illness*	*Alive at hospital discharge without ICU readmission (n=340)*	*Dead at hospital discharge or readmission to the ICU (n=43)*	*P*
Age, mean ± SD	65.4 ± 16.6	71.6 ± 11.9	0.01
Comorbidities, n (%)	255 (75)	42 (97.7)	0.0008
APACHE IV	48.8 ± 23.4	54.7 ± 20.6	0.3
ICU support	141 (41.5)	28 (65.1)	0.003
Predicted mortality rate	8.6	13	< 0.0001

Our study findings, however, differ from a Finnish study from 18 universities and central hospital ICUs which failed to demonstrate an association between after-hour transfer and increased mortality^16^. The difference could be due to differing after-hour definitions and methods for assessing illness severity. The Finnish study adjusted for illness severity using the SAPS II-scores and defined after-hour as 16:00 hours to 08:00 hours. In contrast, our study used APACHE IV score for assessing illness severity and defined after-hour as 20:00 hours to 08:00 hours. Two other studies also did not find any relation between ICU discharge time and mortality. While the first study^10^ stated adequate availability of ICU beds in their setting making premature transfers unlikely and also optimum staf?ng round the clock, the latter^21^ had a different methodological design.

The proportion of night time discharges in our study (28.2%) was higher compared to the mean of 15.3% (range:3.6% to 34.7%) in a recently reported meta analysis^22^. There was no significant difference in the demographic characteristics between patients transferred during daytime and those transferred during after- hours and they were also similar in terms of baseline comorbidities. However, a higher proportion of patients admitted to ICU after surgery from the operation theaters were transferred to ward during daytime. This likely represents a group of elective admissions who were probably kept for post operative monitoring and their transfer planning was therefore relatively straightforward. As opposed to this, patients transferred during after-hours had higher APACHE IV scores with higher predicted mortality, and they were more likely to have sepsis and organ support requirement in terms of mechanical ventilation and/or vasopressors. This constitutes a sicker population of patients which as shown in earlier studies^8,13^, might explain increased risk of mortality associated with transfers during after-hours.

Crude bi-variate analysis showed association of older age, presence of comorbidities, APACHE IV score and organ support (ICU support) with increased hospital mortality. However, after adjustment for these variables, after-hour transfer remained the single independent predictor of hospital mortality. This has also been convincingly demonstrated by Yang et al.^22^ in their meta analysis of fourteen studies which showed night time discharge to significantly impact mortality even after adjustment for disease severity at ICU admission and discharge. Unadjusted analysis in our study also showed a higher ICU readmission rate as well as a longer hospital LOS for patients transferred during after-hours compared to daytime transferred patients. However, both these secondary outcomes did not achieve statistical significance in the adjusted analysis.

Several factors that contribute to a patient's outcome following ICU discharge include comorbidities, age and APACHE IV score not only at time of admission but also at time of transfer, as well as continuing organ support requirement after ICU discharge. The staffing of wards both in terms of manpower and ability to provide continuity of care is of paramount importance. It has been shown that presence of organ dysfunction as well as requirement of higher levels of support following ICU discharge could contribute to higher mortality^22^. Intensivists should thus recognize this high-risk group and exercise caution while transferring such patients from the ICU in after-hours when continuity of care might be compromised. Placing limitations on care and resuscitation measures may also impact outcome of such sicker patients after ICU discharge. Finally in the Indian scenario, financial constraints influencing decisions to transfer patients prematurely from the ICU when a patient would ideally have benefited from 24-48 hours of additional ICU care, is a common phenomenon and can impact patient outcome.

Our study has limitations. The study was observational in nature with retrospective analysis thereby limiting us to information that was already collected in the database. Severity of illness at ICU discharge is an important factor influencing post ICU outcome. Our study did not have this data. This was a single-centered study. Our institute also has unique organizational characteristics such as limited ICU and HDU beds, and limited skilled manpower in after-hours in the ward - both medical and nursing especially for high-risk patients. The scenario from our ICU thus, might not be generalizable to other hospitals where rapid response teams, ICU outreach services or medical emergency teams are available who can provide extension of ICU care to ward patients round the clock. Also limitation of medical treatment is a major subgroup of patients which has been analyzed in almost all prior studies; however, we had few such patients and excluded them from our analysis. Finally, in an observational study, finding an association cannot be interpreted as causation. However, this is the first study from India to study association of ICU transfer time and outcome. The findings from this study may help physicians take decisions on after-hour transfer of patients.

## CONCLUSION

In conclusion, this study has identified and also supports an association between after-hour transfer and mortality of ICU survivors. Further research will likely identify precise factors, linking mortality associated with ICU transfer time and the organizational processes that could be put in place to reduce mortality rates.
